# SARS-CoV-2 3CL-protease inhibitors derived from ML300: investigation of P1 and replacements of the 1,2,3-benzotriazole

**DOI:** 10.21203/rs.3.rs-2880312/v1

**Published:** 2023-05-11

**Authors:** Alice Hooper, Jonathan D. Macdonald, Brenna Reilly, Joshua Maw, Aidan P Wirrick, Sang Hoon Han, A. Abigail Lindsey, Emma G Rico, Todd Romigh, Christopher M Goins, Nancy S Wang, Shaun Stauffer

**Affiliations:** Cleveland Clinic Lerner Research Institute

**Keywords:** 3CLpro, Mpro, SARS-CoV-2, Covid-19, coronavirus

## Abstract

Starting from compound **5** (CCF0058981), a structure-based optimization of the P1 subsite was performed against the severe acute respiratory syndrome coronavirus (SARS-CoV-2) main protease (3CL^pro^). Inhibitor **5** and the compounds disclosed bind to 3CL^pro^ using a non-covalent mode of action that utilize a His163 H-bond interaction in the S1 subpocket. In an effort to examine more structurally diverse P1 groups a number of azoles and heterocycles were designed. Several azole ring systems and replacements, including C-linked azoles, with similar or enhanced potency relative to 5 were discovered (**28**, **29**, and **30**) with demonstrated IC_50_ values less than 100 nM. In addition, pyridyl and isoquinoline P1 groups were successful as P1 replacements leading to 3-methyl pyridyl **36** (IC_50_ = 85 nM) and isoquinoline **27** (IC_50_ = 26 nM). High resolution X-ray crystal structures of these inhibitors were utilized to confirm binding orientation and guide optimization. These findings have implications towards antiviral development and preparedness to combat SARS-like zoonotic coronavirus outbreaks.

## Introduction

**Figure 1:**
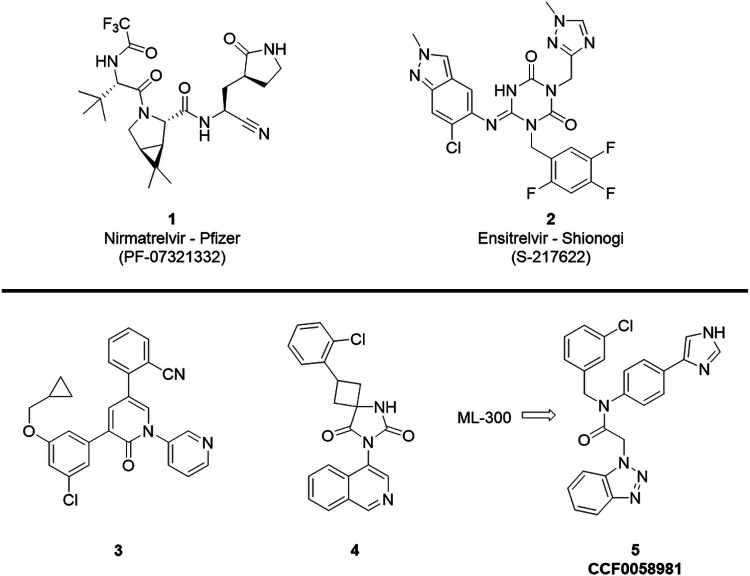
Clinical agents and pre-clinical non-covalent SARS-CoV-2 3CL^pro^ inhibitors: **1** (Nirmatrelvir)[8], **2** (Ensitrelivr)[13], **3** [10], **4 [**15**]**, and ML300 derived **5** (CCF0058981) [17].

The Severe Acute Respiratory Syndrome Coronavirus 2 (SARS-CoV-2) has been one of the greatest health threats in a generation with nearly 7 million deaths worldwide since Coronavirus disease (COVID-19) took hold [1]. Prior short-lived and overall much less deadly outbreaks include SARS-CoV (2003) and MERS (2012) [2]. Despite promising vaccination efforts and status, antiviral therapeutics are considered essential to reduce symptoms in infected patients, prevent hospitalizations, protect high risk immunocompromised populations, and an obligatory investment to enhance preparedness for future outbreaks. Among the approaches to target SARS-CoV-2, inhibitors of the chymotrypsin like main protease (3CL^pro^) have been a subject of intense focus due to the vital role it plays in viral replication [3]. Peptidic covalent inhibitors have been a major focus, which dates back to the initial efforts after SARS-CoV-1 in 2003 [4]. In 2021, Pfizer received emergency use authorization for the use of their covalent SARS-CoV-2 3CL^pro^ inhibitor, PF-07321332 or nirmatrelvir (Figure 1, **1**) for the treatment of acute disease. As impressive and welcome this early success has been to clinical care major efforts continue toward the development of improved potent inhibitors in order address limitations of this therapy. For example, nirmatrelvir requires co-dosing with ritonavir to boost its exposure to sustain plasma concentrations sufficiently above the cellular EC_90_ in order to provide adequate target coverage. Drawbacks of related peptide-based covalent inhibitors include low membrane permeability and the required reactivity derisking inherent from the covalent warhead [5-7]. Thus, next generation inhibitors have the opportunity to offer significant improvement provided they can achieve desirable PK and potency. These parameters must be set with a high bar in mind in order to achieve clinically meaningful antiviral efficacy and a desirable safety profile for potential prophylactic applications [8].

With a renewed focus on such efforts a number non-peptidic, non-covalent 3CL^pro^ inhibitors have appeared in the last two years to offer new starting points for coronavirus antiviral efforts [9-12]. The structure of one clinical candidate by Shionogi in partnership with Hokkaido University,[13] known as Ensitrelvir (**2**), was approved in Japan in November of 2022. In preclinical models of antiviral activity **2** demonstrated an EC_50_ of 370 nM in a TMPRSS2 VeroE6 model of SARS-CoV-2 infection. In 2021 a promising perampanel-derived non-covalent series of inhibitors of 3CL^pro^ exemplified by **3** was disclosed by the Jorgensen and Anderson groups [10, 14], achieving an antiviral EC_50_ of 980 nM in a SARS-CoV2 plaque reduction assay. In early 2022, the Carlsson team published excellent work disclosing the broad-spectrum inhibitor **4** derived from an ultralarge virtual screening campaign which utilized MLPCN probe ML188 as a starting ligand [15]. In SARS-CoV-2 infected Huh7 cells in a CPE-based assay format compound **4** displayed antiviral activity comparable to that of **1** with an EC_50_ of 110 nM. Non-covalent 3CL^pro^ inhibitors **3** and **4** bear a related 3-pyridyl or isoquinoline ring system as a P1 group, the former first identified from SARS-CoV-1 probe ML188 [9] and then later by the COVID Moonshot program [16]. Previously, our group described the development of the benzotriazole based P1 inhibitor **5** [17] from a structure-guided optimization of ML300. In a SARS-CoV-2 enzymatic assay **5** inhibited SARS-CoV-2 3CL^pro^ with an IC_50_ of 68 nM and in a VeroE6 based plaque reduction assay afforded an EC_50_ of 558 nM.

Despite initial promising findings for lead compound **5** there were several shortcomings to be addressed including the modest cellular activity. In addition, the series suffered high metabolic clearance and CYP inhibition. Metabolite identification studies highlighted metabolic soft-spots on the *N*-benzylic portion of the molecule and up to one-third abundance of metabolism occurring within the P1 benzotriazole ring system [17]. Herein, we describe findings from a more extensive optimization of the P1 and interactions within the S1 sub-pocket, including the first X-ray structures of carbon-linked heterocycle based inhibitors within the ML300 series bound to SARS-CoV-2 3CL^pro^ as replacements of the *N*-linked 1,2,3-benzotriazole moiety. \

**Figure 2:**
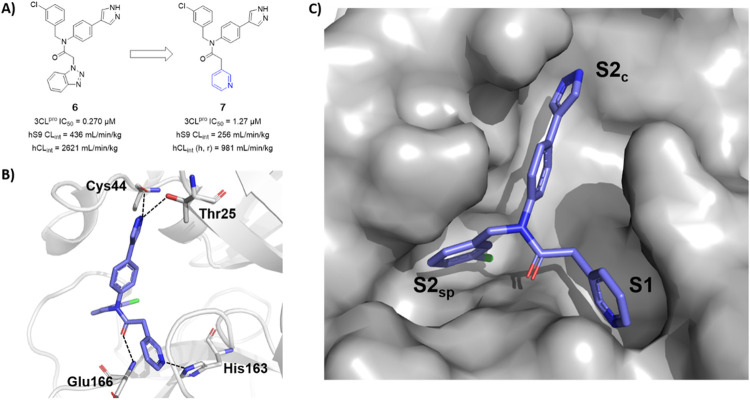
a) Evolution and profile of previously reported benzotriazole **6** and pyridine **7** (b) X-ray co-crystal structure of **7** in a complex with SARS-CoV-2 3CL^pro^ (PDB entry 7TEK) solved at 2.2 angstroms, (c) Solvent accessible surface display inhibitor **7** within sub-pockets S2_sp_, S2_c_ and S1.

We began by examining the crystal structure of the SARS-CoV-2 3CL^pro^:ML300 complex (PDB: 7LME), and utilized molecular docking and structure-guided design to incorporate replacements of the 1,2,3-benzotriazole into P1 [17]. A hydrogen bonding interaction with His163 is a key feature of ML300 and other published sub-micromolar 3CL^pro^ inhibitors, both covalent and non-covalent [17, 9, 18, 11, 10] which defines the S1 subpocket. The removal of atoms necessary to achieve this anchoring interaction is significantly detrimental to binding affinity. As shown in Figure 2, this initial effort led to pyridine **7**. Compound **7** as described previously [17], represents a 10-fold reduction in potency versus the benzotriazole **6**. In contrast, in human microsomes and S9 fractions pyridine congener **7** demonstrated a two-fold reduction in intrinsic clearance metabolism, albeit still high scaled to hepatic blood flow. To confirm the predicted orientation and binding pose of **7** and understand approaches to improve potency we obtained a high resolution X-ray structure bound to SARS-CoV-2 3CL^pro^. Indeed, inhibitor **7** occupies a similar orientation and presentation of the P2_c_ and P2_sp_ groups compared to prior inhibitors in the series, utilizinga pyrazole and cholorophenyl ring, respectively. Importantly, the pyridine nitrogen acts a suitable hydrogen bond acceptor towards His163 with an interatomic distance of 2.8 angstroms. Other putative hydrogen bond interactions include the P2_c_ pyrazole nitrogens within 3.0 angstroms of Cys44 backbone carbonyl and Thr25 side chain. In addition, there is a highly conserved 3.0 angstrom interaction between with the Glu166 backbone NH and the anilido amide oxygen. Since **7** displayed an encouraging yet non-optimal profile (MW 402, LE = 0.27) and a crystal structure bound to 3CL^pro^ was in hand, we performed a series of docking studies to design and prioritize analogues that incorporate a hydrogen bond acceptor to mimic His163 interaction and potential secondary interactions at the *N*-termini loop of the oxy anion hole region (Leu141-Ser144).

Table 1 below depicts SARS-CoV-2 3CL^pro^ inhibition structure-activity data focusingon monocyclic P1 modifications with compound **7** as a comparator.

Pyridine, **7** and pyrimidine, **8** maintain modest IC_50_ values against 3CL^pro^, 1.27 μM and 3.44 μM respectively. Initial exploratory SAR in the S1 pocket involved replacing P1 heterocycles with pyrazine, **9**, resulting in a >2-fold loss in potency at 6.56 μM and the pyridazine, **10**, which maintained micromolar activity, within 4-fold of parent compound **7**. Replacement of a 6-membered heterocycle with a 5-membered 1,2,3-triazole, **11**, and thiazoles **12**, **13** and **14** showed no improvement vs. **7**. Compound **11** is particularly surprising considering the 1,2,3-benzotriazole **6** has an IC_50_ of 270 nM representing a 30-fold loss relative to **11**. This observation may in part be due to a combination of an electronic effect of the bicyclic ring and/or based on the structural data a space filling property of the extended ring within the P1 where van der Waals contacts with backbone loop residues Lue141 and Asn142 engage in favorable contacts. Heterocycles substituted with either a small methyl (1**5**), or larger phenyl group (**16** and **17**) resulted in either a significant 20-fold loss or were immeasurable. Finally, saturation of the heterocycle in the form of lactam **18**, a key motif utilized in peptidomimetic 3CL^pro^ inhibitor discovery programs, most notably by Pfizer in *nirmatrelvir* [18], also resulted in no measurable inhibition when tested in racemic form at 100 mM as the top concentration.

One common challenge observed during optimization of 3CL^pro^ inhibition has been differential SAR as a result of the flexibility within loop regions of the active site. This is particularly noted within ML300 series which undergoes a reorganization of the S2 subpocket to accommodate both the *N*-benyzl P2_sp_ and the azole P2_c_ moiety [17]. In an effort to find more texture in SAR to suitably assess P1 replacements we replaced the pyrazole of **7** with an often more potent imidazole and further addition of a *meta*-fluoro substitution on the P2_sp_ phenyl ring to afford **19** (Table 2). This amalgamation of improvements leads to an overall ~8-fold improvement of IC_50_ to 0.15 μM whilst also improving ligand efficiency [19-21]. With **19** as a sub-micromolar starting point containing the C-4 imidazole as the P2c group we revisited prior P1 modifications (Table 2, left column). In addition, in context of the 3-fluoro-5-chloro substitution a number of bicyclic P1’s (Table 2, right column) were examined, including a number of benzotriazolyl P1’s to further improve inhibitory activity relative to **5**.

Direct comparison of the 6-membered heterocycles **7** and **8** with **19** and **20** demonstrates the impact of the P2-channel and P2-subpocket replacements, as the pyridine and the pyrimidine now have improved IC_50_’s of 150 nM and 530 nM, respectively. The trend continues with pyrazine **21** showing a greater than 3-fold increase in potency when compared to **9** and similarly pyridazine **22** in the imidazole series has an IC_50_ value of 520 nM, an 8-fold improvement vs.pyrazole **10**. Within the 5-membered P1 analogues trends noted within the pyrazole series generally carry over within this series. For example, the 1,2,3-triazole **23** displays a 20-fold increase in inhibition; unfortunately once again saturated gamma lactams, such as **24-26** as various regioisomers, remain inactive.

Utilizing X-ray structural data for 7 and previously published structures containing the bicyclic 1,2,3-benzotriazole,[17] a series of bicyclic heterocycles were synthesized to more effectively fill the S1 pocket (27-34). Pleasingly, direct bicyclic isoquinoline 27 demonstrated a greater that 6-fold improvement in IC_50_, compared to the monocyclic pyridine 19 with an IC_50_ of 26 nM. Extension to the isoquinoline 27 retains LE per heavy atom, but a reduction in LipE is observed (2.65 vs. 2.24), suggestive this change is positive for potency but not optimal for physicochemical properties. The 1,2,3-benzotriazole 28 is nearly equipotent to 27 representing a novel C-linked P1 group within the series. Alternate fused 5,6-ring azole analogues containing a triazolo or imidazo pyridine system to retain a C-linked aetamide were synthesized (29-32). For the triazolo[4,3-*a*]pyridine 29 the IC_50_ remained sub-micromolar with an IC_50_ of 83 nM, while removal of one nitrogen to the imidazo[1,2-*a*]pyridine 30 demonstrated statistically similar potency at 97 nM. Per X-ray crystallography and modeling, it is clear that the *N*1 nitrogen of the triazolo[4,3-*a*]pyridine structure is important, and the *N*2 nitrogen does not appear to be affecting binding affinity significantly; however, the overall SAR suggests 1,2,3-benzotriazole is slightly more favorable compared to 1,2,4-isomer. Introduction of a fluorine in the 5-postion of 1,2,4-triazole analogue 31 was not deleterious to the *N*1 hydrogen-bond acceptor (IC_50_ of 106 nM). P1 replacement with a 6-methyltriazolo[4,3-*b*]pyridazine 32 resulted is a significant 5-fold drop in potency relative to 28. Switching the key hydrogen-bonding accepting heteroatom from nitrogen to oxygen, in the form of benzisoxazole, 33, resulted in a significant loss of potency (>20-fold). Finally, although an improvement by demonstrating measurable activity compared to monocyclic comparators 18 and 25, indolin-2-one 34 maintained only weak micromolar inhibition (IC_50_ = 6.24 mM).

In light of the comparable potency of 27 and 28 we obtained a high resolution X-ray structure of isoquinoline 27 to confirm it’s orientation within the S1 pocket. Indeed, a number of potent non-covalent inhibitors bearing an isoquinoline have been leveraged to target SARS-CoV-2 3CL^pro^ [15, 16]. Shown below in Figure 4 is an overlay comparing the orientation of 27 and 7.

**Figure 3:**
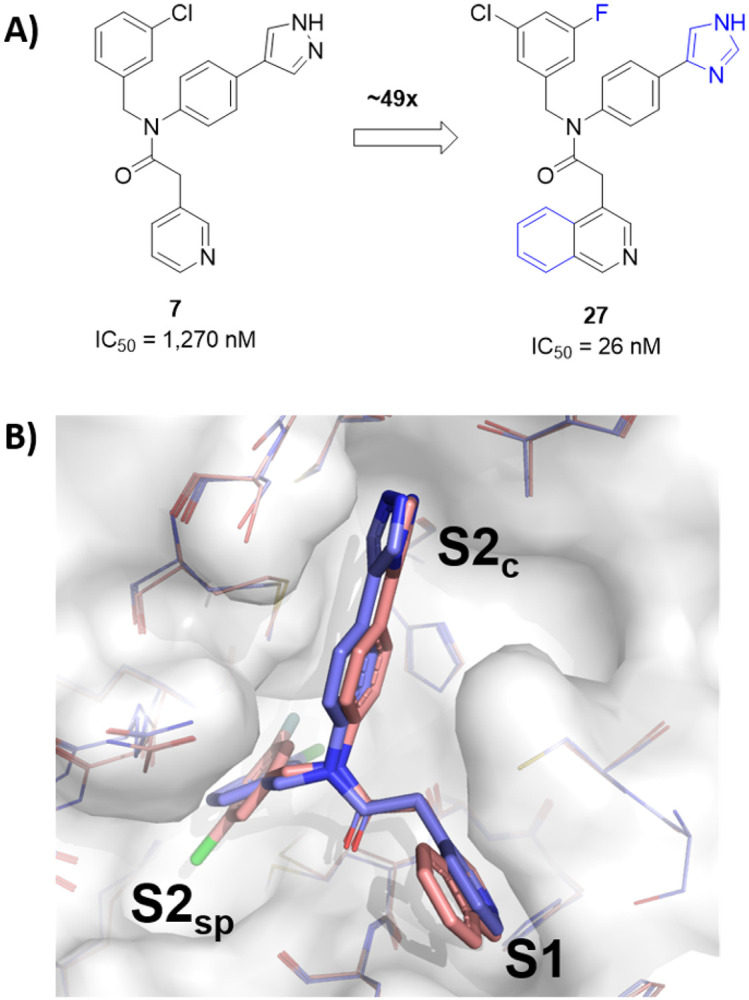
(a) Pyridyl based inhibitors **7** and **27**, (b) comparison of X-ray co-crystal structures of **7** (light purple sticks, PDB entry 7TEK) and **27** (salmon sticks, PDB entry 7TEL) in complex with SARS-CoV-2 3CL^pro^ (surface from 7TEL, select residues show as lines).

The co-crystal complex of 27 (Figure 3) reveals essentially identical positioning of the isoquinoline nitrogen relative to the pyridyl nitrogen of 7 as expected. There are obvious differences within the P2_c_ and the P2_sp_, including a chlorine halogen preference deeper within the P2_sp_ when the ring system is no longer substituted with fluorine. This is the subject of a more in depth disclosure beyond the scope of this report. As discussed above and as revealed from the structures disclosed the benzo ring of the isquinoline appears to modestly impact electronics and/or provide a subtle steric bulk interaction with Asn142. In order to improve LipE of 27 relative to 19 we asked whether we could modulate interactions in this P1 groove near Asn142 with simpler substitutions. With this in mind, a handful of substituted pyridines were designed in the context of the study in Table 2 to assess whether potency could be maintained or improved while favorably impacting calculated physicochemical properties.

Interestingly, introduction of a fluorine in the 3-position of the pyridine (**35**) resulted in a loss in primary potency in comparison to both the unsubstituted pyridine **19** and the isoquinoline **27**. However, exchanging the 3-fluoro to a 3-methyl **36** led to 2-fold improvement in the IC_50_, 217 nM to 84 nM, respectively, but also a much improved LE and LipE. Unfortunately, this trend is not maintained with larger 3-cyclopropylpyrine derivative **37** as both LE and LipE trend in opposite direction. Finally, we introduced 4-substituted pyridines (**38-40**) which resulted primarily in higher IC_50_ values in comparison to the unsubstituted pyridine **19**; however, simple methyl derivative **38** was equipotent to **19**. Overall findings from this limited study suggests SAR around 3 and 4-position of the P1 pyridyl ring system is narrow in scope, consistent with the structural information.

The findings from this P1 study offer new directions to further optimize antiviral activity and metabolic stability to develop nanomolar inhibitors against SARS-CoV-2 and future variants. Details from these efforts will be the subject of a future disclosure. In summary this study led to an enhanced understanding of P1 structural diversity and SAR within the ML300 series. In addition, molecules were identified with good potency, including first examples in the series with IC_50_ values under 30 nM and evidence of improved calculated properties. X-ray crystal structures of pyridine and isoquinoline based inhibitors **7** and **27** provide a basis for further optimization of these new P1 substructures. These findings have implications towards antiviral development to combat current and future SARS-like zoonotic coronavirus outbreaks.
